# Supplementary material for: Occupational exposure to dust and respiratory symptoms among Ethiopian factory workers: A systematic review and meta-analysis
Source: PLoS One. 2023 Jul 21;18(7):e0284551. doi: 10.1371/journal.pone.0284551 (PMC10361507; doi:10.1371/journal.pone.0284551)
Supplement: S1 Table — (DOCX) [file pone.0284551.s001.docx]

**S1 Table.** **Summary of search results for the PubMed, Google Scholar, Science Direct, African Journals Online and Web of science**

| Searched databases | Search terms | Number of studies |
| --- | --- | --- |
| Google scholar | prevalence or magnitude of respiratory symptoms or pulmonary symptoms and associated factors or determinants among Ethiopian factory or industry workers | 1430 |
| PubMed | ((((("prevalence"[MeSH Terms] OR "prevalence"[Text Word] OR "magnitudes"[All Fields]) "epidemiology"[MeSH Terms] OR Epidemiology[Text Word] AND "respiratory symptoms"[Text Word] AND "risk factors"[MeSH Terms] OR Risk factors[Text Word] AND "occupational exposure"[MeSH Terms]) OR "occupational exposure"[Text Word]) AND "textile industry"[MeSH Terms]) OR "textile industry"[Text Word] OR "flour"[MeSH Terms] OR "flour"[Text Word] OR "wood"[MeSH Terms] OR wood[Text Word] OR cotton [Text Word] OR "cement"[Text Word] OR "paper"[MeSH Terms]) OR Grain[Text Word] AND "Ethiopia"[MeSH Terms] | 125 |
| From other databases* |  | 40 |
| From university databases |  | 1 |
| Total retrieved articles |  | 1596 |
| Studies included in final analysis |  | 15 |

* Science Direct, African Journals Online and Web of science
