# Supplementary material for: Occupational exposure to dust and respiratory symptoms among Ethiopian factory workers: A systematic review and meta-analysis
Source: PLoS One. 2023 Jul 21;18(7):e0284551. doi: 10.1371/journal.pone.0284551 (PMC10361507; doi:10.1371/journal.pone.0284551)
Supplement: Attachment — Submitted filename: Response to reviewers.docx [file pone.0284551.s003.docx]

**Point by point response**

| S.No | **Questions or comments** | **Responses** |
| --- | --- | --- |
|  | **Reviewer #1** |  |
| 1 | Paragraph 6 in the introduction section “The prevalence of occupational…….to 69.8% wood factory workers” has some overlapping/repetitive information with paragraph 2 in the discussion section “According to the findings…………contributing factors to the discrepency.” Either paragraph can be rephrased to avoid it. | Thank for your comment. we have revised our manuscript as per your comment |
| 2 | Paragraph 7 in the introdcution section starts with “However, the research findings are inconsitent.” Which research findings are inconsistent and why do the authors think so? | Thank you a lot. We revised it. It is necessary to pool the prevalence of respiratory symptoms and associated factors at the factory level due to variations in findings across previously existing primary studies. |
| 3 | In the last paragraph of introduction section, the authors should focus and elaborate on the scope of this manuscript compared to previous studies i.e. pooled estimates of prevalence in different industries vs. industry specific prevalence in previous studies. | Thank you very much for your advice. Previous systematic reviews and meta analyses pooled some of the studies conducted outside of industries or outdoor environments, for example, among street sweeping workers. As a result, it is not the correct method because the exposure status and working environment differ in the outdoor and indoor environments. Furthermore, dust control measures differ in the indoor and outdoor environments. Engineering control measures, for example, are inapplicable in an outdoor environment. As a result, estimating pooled prevalence specifically among factory workers was critical in order to implement appropriate prevention and control measures to improve worker and factory owner productivity. Therefore, we conducted this systematic review and meta analysis of studies. |
| 4 | Research question should be within the text in the introduction section. | Thank you. We replaced the research questions with the study's objective. |
| 5 | It is better to put table 1 in the supplementary information. | Thank you for your constructive feedback. Table 1 was added as supplementary information. |
| 6 | It is better to discuss the limitations in the discussion section rather than having a separate section. | Thank you. We revised accordingly. |
| 7 | Please revise the manuscript for grammatical/language corrections. For example, there should be no “and” in the first line in the result of the abstract “….studies that met inclusion criteria and were included….” Another example, there is no verb in the last line of fifth paragraph of introdcution section “…The reported rspiratory….magnitude…..to factory/industry”. | Thank you a lot. We revised the the whole manuscript to correct grammatical and language errors. |
|  | **Reviewer #2** |  |
| 1 | I have concern with previous published works, which is more convenient than yours. Also, you need to finalize the statistical analysis in the way all the works presents and graph them accordingly. | Thank for your comment. We changed the entire document in the revised version. This study differs from previous ones in that it focuses primarily on factory workers. However, studies conducted among outdoor workers were included in previous systematic reviews and meta analyses. I believe that including studies conducted among street sweeping workers (outdoor environment) was not the correct approach because exposure status, type of dust generated, and control measures implemented were completely different in indoor and outdoor work environments. As a result, combining outdoor and indoor workplace prevalence is not the best approach. We performed various statistical analyses such as meta regression, sensitivity analysis, and Egger's test. |
