# Supplementary material for: Occupational exposure to dust and respiratory symptoms among Ethiopian factory workers: A systematic review and meta-analysis
Source: PLoS One. 2023 Jul 21;18(7):e0284551. doi: 10.1371/journal.pone.0284551 (PMC10361507; doi:10.1371/journal.pone.0284551)
Supplement: Attachment — Submitted filename: Response to reviewers.docx [file pone.0284551.s004.docx]

**Point by point response to reviewers**

| S.No | **Questions or comments** | **Responses** |
| --- | --- | --- |
| 1 | The authors should provide a PRISMA Checklist to enhance the transparency and completeness of their systematic review. | Thank for your comment. We provided a PRISMA Checklist as supporting information as per your comment |
| 2 | The article's methodology should include a clear definition of respiratory symptoms. | Thank you very much. In the revised version of the manuscript, we defined respiratory symptoms |
| 3 | The results of the selection process should be reported as usual for this type of study. Specifically, the study's selection process from L170 to 175 should be presented in the results section | Thank for your comment. In the revised version, we presented the selection process in the results section. |
